# Matching Biological Mesh and Negative Pressure Wound Therapy in Reconstructing an Open Abdomen Defect

**DOI:** 10.1155/2014/235930

**Published:** 2014-03-19

**Authors:** Fabio Caviggioli, Francesco Maria Klinger, Andrea Lisa, Luca Maione, Davide Forcellini, Valeriano Vinci, Luca Codolini, Marco Klinger

**Affiliations:** ^1^University of Milan, School of Plastic Surgery, Plastic Surgery Department, Multimedica Holding S.p.A, Via Milanese 300, Sesto San Giovanni, 20099 Milan, Italy; ^2^University of Milan, School of Plastic Surgery, Department of Translational Medicine (BIOMETRA), Humanitas Clinical and Research Center, Via Manzoni 56, Rozzano, 20089 Milan, Italy; ^3^Università degli Studi di Milano, Dipartimento di Biotecnologie Mediche e Medicina Traslazionale (BIOMETRA), IRCCS Istituto Clinico Humanitas, U.O. Chirurgia Plastica, Via Manzoni 56, Rozzano, 20090 Milan, Italy

## Abstract

Reconstruction of open abdominal defects is a clinical problem which general and plastic surgeons have to address in cooperation. We report the case of a 66-year-old man who presented an abdominal dehiscence after multiple laparotomies for a sigmoid-rectal adenocarcinoma that infiltrated into the abdominal wall, subsequently complicated by peritonitis and enteric fistula. A cutaneous dehiscence and an incontinent abdominal wall resulted after the last surgery. The abdominal wall was reconstructed using a biological porcine cross-linked mesh Permacol (Covidien Inc., Norwalk, CT). Negative Pressure Wound Therapy (NPWT), instead, was used on the mesh in order to reduce wound dimensions, promote granulation tissue formation, and obtain secondary closure of cutaneous dehiscence which was finally achieved with a split-thickness skin graft. Biological mesh behaved like a scaffold for the granulation tissue that was stimulated by the negative pressure. The biological mesh was rapidly integrated in the abdominal wall restoring abdominal wall continence, while the small dehiscence, still present in the central area, was subsequently covered with a split-thickness skin graft. The combination of these different procedures led us to solve this complicated case obtaining complete wound closure after less than 2 months.

## 1. Introduction

Abdominal wall defects caused by trauma or surgery are a common surgical problem to solve. Reconstruction of complicated abdominal dehiscence is a challenging condition for both general and plastic surgeons and several techniques have been described.

Predisposing factors for lack of skin closure include inadequate local fascial and muscular layers due to prior tissue loss, muscle denervation or vascular insufficiency due to prior irradiation or infection, wound infection, obesity, chronic pulmonary disease, malnutrition, sepsis, anemia, corticosteroid dependency, and/or concurrent malignant process. Biological abdominal mesh is a new possible therapeutic approach in patients with open abdomen that increased mesh abdominal repair indications in patients with abdominal wall defects [1].

In some cases, when abdominal continence has been obtained but a wound dehiscence persists, Negative Pressure Wound Therapy (NPWT) could be a therapeutic option to obtain wound closure [2]. This treatment involves application of topical negative pressure to the open wound. It is an innovative treatment which increases the number of cases that can now be solved [3].

We report a case where we applied NPWT on a biological abdominal mesh in order to reduce wound dimensions over the implant and to prepare wound bed for a split-thickness skin graft which was subsequently used to definitely close wound dehiscence.

This study widens the possible application of NPWT confirming its therapeutic role also if applied on biological mesh in complicated patients.

## 2. Case Presentation

We report a case of a 66-year-old man who was diagnosed in 2010 of sigmoid-rectal adenocarcinoma.

At the moment of the diagnosis CT scan revealed a bulky mass (8 × 7 cm) in the sigmoid colon with no cleavage plane with the bladder and the abdominal wall and perilesional fat micronodulations. First operation was performed in another hospital. In that occasion the cancer was entirely removed and colostomy in the transverse colon was created. Local resection was followed by chemoimmunotherapy since there were no more signs of progression.

On December 2011 the patient came to our hospital. The cancer remained focal with no signs of metastasis. CT scan, indeed, showed a voluminous solid mass localised in the sigmoid colon that infiltrated the bladder and the abdominal wall including fascia and muscles up to the subcutaneous tissue. General surgeons proceeded with an anterior pelvic* exenteratio *removing the sigmoid colon en bloc, part of the ileum, the bladder, and the area of the abdominal wall infiltrated by cancer. Abdominal wall was reconstructed with a monofilament abdominal mesh in the lower abdominal quadrants, together with a ureterocutaneous stomy.

Afterwards, the presence of an enteric fistula caused a retroperitoneal pool and another surgery was needed. A relaparotomy was performed with abdominal mesh removal, enterorrhaphy, and an extended viscerolysis. Abdominal wall was finally closed with a macroporous polypropylene mesh.

Four days later signs and symptoms of peritonitis were diagnosed and the patient underwent a third surgery: the mesh was removed for the second time and another explorative laparotomy was made, followed by drainage of peritoneal cavity, viscerolysis, and creation of a lateral ileostomy in the left iliac* fossa*. Primary closure of the abdomen was not possible to achieve and an open abdomen negative pressure therapy system was applied over the peritoneal organs with a pressure of −125 mmHg in order to reduce edema. Dimensions of cutaneous dehiscence were, at the moment, 16 × 10 cm.

**Figure 1:**
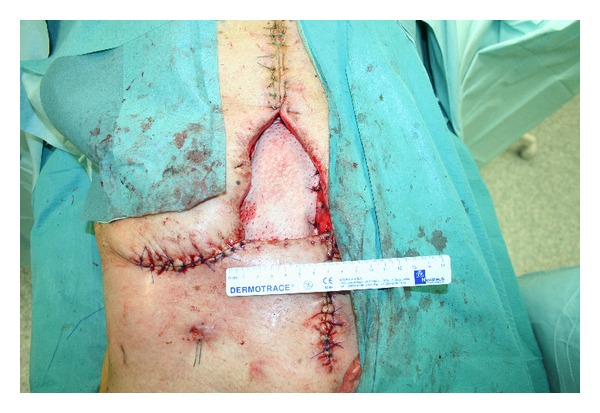
Application of biological mesh over intestinal loops to recreate abdominal wall.

Three days later, a further valuation showed an improved clinical presentation of intestinal loops. The abdominal wall was then reconstructed using a biological porcine cross-linked mesh, Permacol (Covidien Inc., Norwalk, CT), of 20 × 15 cm overlapped and fixed to the fascial edges of the incision using nonabsorbable stitches. Biological mesh was used for its lower recurrence rates if compared with allograft and other biological meshes [4]. An advancement of the cutaneous tissue was made in order to reduce dehiscence as much as possible, obtaining a final dimension of 12 × 7 cm (Figure 1).

Postoperative period was characterized by intestinal motility resumption through the ileostomy and conservation of renal function thanks to the ureterocutaneous stomy; oral alimentation was reintroduced. Patient was then transferred to our department.

**Figure 2:**
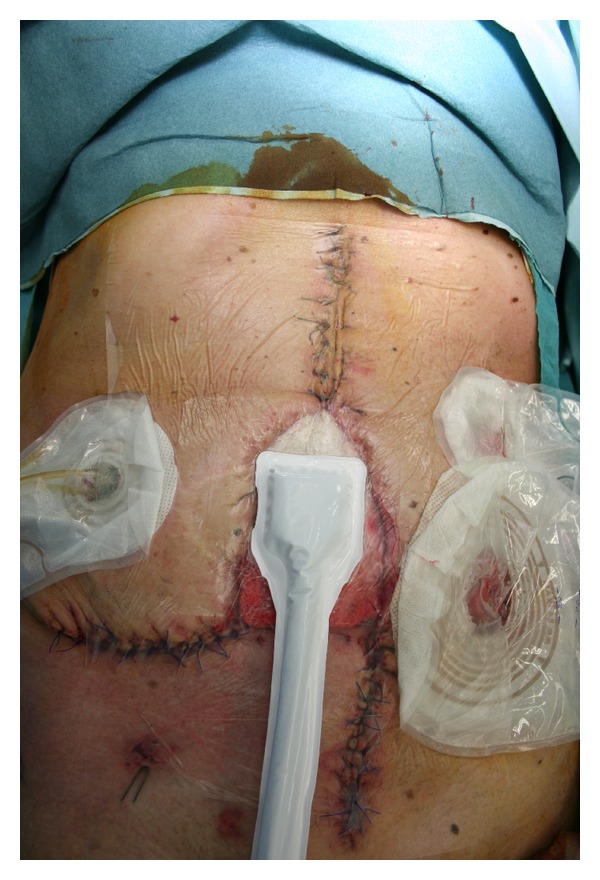
NPWT applied over the biological mesh.

He presented a 12 × 7 cm abdominal dehiscence which could not be closed neither directly nor with local advancement flaps because of excessive tension on wound edges. Moreover, patient clinical conditions made adoption of myocutaneous regional or distant flaps contraindicated. We subsequently considered NPWT as the best therapeutic option in order to reduce wound dimensions and to obtain secondary closure. For the first period negative pressure was set at −70 mmHg and was renewed twice a week (Figure 2). We decided to adopt a lower pressure at the beginning as we were not confident to apply it to a biological mesh.

After four days of treatment the wound showed signs of local infection. Swab revealed the presence of* Pseudomonas Aeruginosa *and* Corynebacterium species*, two of the most common nosocomial pathogens in our experience. The patient was then treated with Ciprofloxacin (500 mg/bid) and Meropenem (1 g/tid). Locally the wound was disinfected with chlorhexidine and treated with a silver antimicrobial barrier dressing combined with NPWT. Infection was controlled and after 10 days of systemic and local therapies we obtained a negative swab for both pathogens.

Two weeks after NPWT application and once local infection has been controlled patient was discharged. At that point he still presented a 6 × 4 cm dehiscence of the abdominal wall. Moreover granulation tissue have been started to cover biological mesh and we decided to increase the negative pressure to −90 mmHg in order to further improve granulation tissue growth and to obtain a total coverage of the biological mesh.

**Figure 3:**
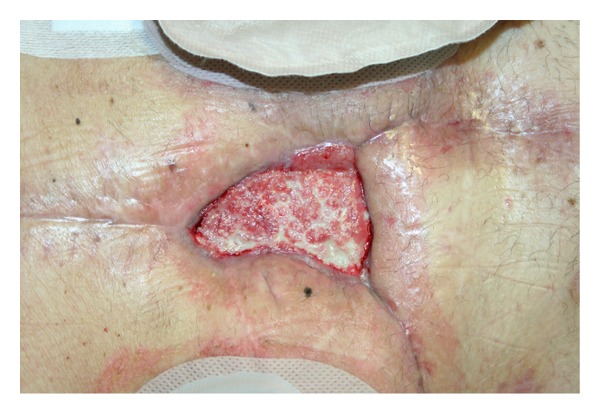
Abdominal dehiscence after 28 days of NPWT.

Patient was therefore followed on outpatient basis twice a week. Wound dimension progressively decreased and granulation tissue flourished (Figure 3).

**Figure 4:**
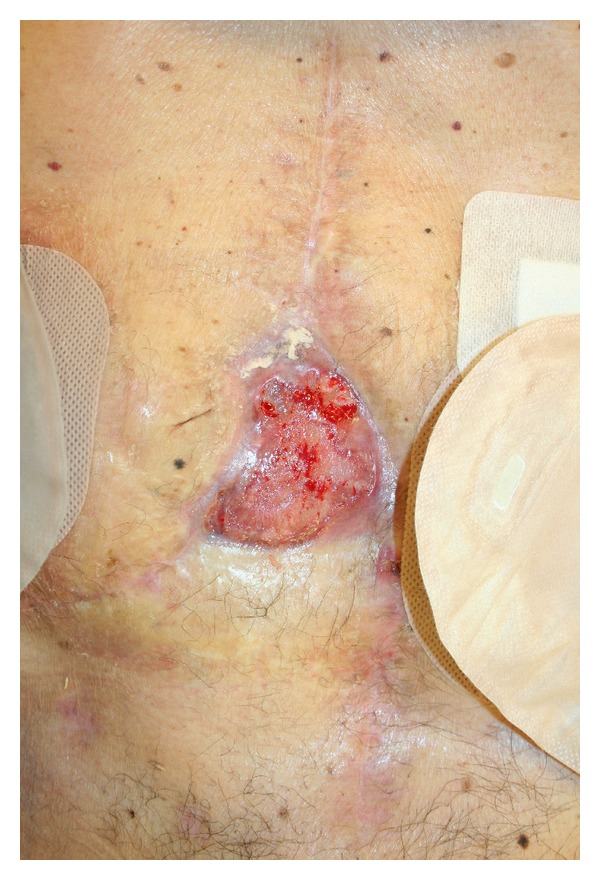
The final abdominal dehiscence after 43 days of NPWT.

NPWT was removed after a total therapeutic period of 43 days. During this period negative pressure was set at −70 mmHg for a total of 14 days and −90 mmHg for the remaining period. Our plan was to further increase negative pressure but the good clinical response allowed us to reduce time of NPWT. During all therapeutic period we adopted a polyurethane foam as dressing. Wound dehiscence starting dimensions were of 12 × 7 cm while, at the end of treatment, the final dimension was 3 × 5 cm (Figure 4).

After NPWT removal wound bed was medicated with alginate and silver sulfadiazine for the following 14 days twice a week in order to prepare it for the final reconstructive step.

When biological mesh was completely overlaid with granulation tissue, the remaining loss of tissue in the lower abdominal area was closed with a split-thickness skin graft taken from the left thigh. The graft takes roots perfectly allowing total closure of abdominal dehiscence after 55 days from patient transferral to our department. No recurrence and local complications occurred with a postoperative follow-up of 6 months.

## 3. Discussion

Colon cancer is the second most frequent neoplasm in the Italian population. However colon carcinoma very rarely presents with subcutaneous, retroperitoneal, or abdominal wall abscess. Abscess of the anterior abdominal wall developing as a result of direct invasion and perforation of the colon by cancer has been rarely described [5–7]. Our patient's tumor was deeply penetrating into the abdominal wall and created an enterocutaneous fistula. This condition requires a wide incision of all involved structures, especially if no signs of lymph nodes metastasis are present [7]. This procedure creates a large defect in the abdominal wall and primary closure is rarely possible, because it leads to high tension in wound edges. This condition, together with the high risk of infection, explains why treating an open abdomen is a serious and challenging problem for both general and plastic surgeons.

Many techniques have been described to reconstruct the abdomen wall but, if complications and reoperation occurs, options for deep abdominal layers reconstruction became progressively fewer. They include direct tissue closure, prosthetic mesh, local advancement or regional flaps, distant flaps, or combined flap and mesh. In particular regional flaps are the rectus abdominis flap and the external oblique flap while distant flaps options include the latissimus dorsi, tensor fascia lata (TFL), and rectus femoris muscles [8].

Porcine acellular dermal collagen implants are made of a biologic material derived from processing porcine dermis cross-linked with diisocyanate [9]. Some authors showed that PADCI induces a milder inflammatory response and lesser formation of adhesions [10]. It has less propensity to infection, erosion, extrusion or rejection_,_ intra-abdominal adhesion, and fistula formation [4]. As they reduce number of side effects, these biological meshes are a favourable choice in pluricomplicated cases when the patient has already underwent many operations and complications. In pluricomplicated cases synthetic meshes should not be used because the rate of recurrence is too high especially due to peritonitis, as in our patient.

Biological mesh is a new frontier in treating complicated open abdomen wounds and clinical evidences are progressively increasing in clinical practice.

On the other hand, NPWT has already been shown to heal complicated abdominal dehiscence.

It works in open abdomen providing mechanical containment of abdominal viscera, third space fluid loss estimation, and prevention of intestinal fistula and infection while in abdominal dehiscence it stimulates granulation tissue growth, removal of exudates, and promotion of neoangiogenesis.

To improve infection control, if clinical condition requires, silver dressing could be added to both polyurethane foam and dressing gauze as in our clinical report.

In our case, we decided to adopt NPWT in a patient who presented an abdominal dehiscence in order to reduce time of hospitalization and to obtain total abdominal wall closure.

NPWT could be managed on outpatient basis; in this way time of hospitalization was reduced together with the nosocomial infection possibility, decreasing costs and improving the tolerability of the procedure.

The association of NPWT and biological mesh implant prevented us to adopt other reconstructive procedures as regional or distant flap which could be much more invasive in our complicated patient.

In our experience the biological mesh behaved like a scaffold for granulation tissue which growth was stimulated by negative pressure therapy allowing a final closure with a split-thickness graft. The two procedures seem to combine perfectly together.

The mesh was rapidly integrated within the abdominal wall, restoring abdominal wall continence. Our clinical case shows that NPWT use is not contraindicated if a biological mesh is present and exposed; on the contrary NPWT could reduce hospitalization time with a decreased rate of complications.
